# The theory of psychic quanta: a quantum model for the unity of individual consciousness

**DOI:** 10.3389/fpsyg.2026.1792921

**Published:** 2026-05-21

**Authors:** Andrea Tallarico

**Affiliations:** Independent Researcher, San Donato Val di Comino, Italy

**Keywords:** consciousness field, IIT, NDE, Orch-OR, panpsychism, past-lifememories, psychic quanta, quantum consciousness

## Abstract

The Theory of Psychic Quanta (TPQ) postulates the existence of a universal non-local psychic field whose quantized excitations—termed *informational quanta (analogous to qubits)*—anchor to coherent brain systems to generate individual consciousness as a phenomenological unit. According to this model, the brain does not produce consciousness in an emergentist sense; rather, it acts as a bidirectional biophysical interface that stabilizes the informational quantum without generating it. This reciprocal interaction maintains experiential coherence from biological birth until the cessation of brain activity, at which point the quantum disanchors and reintegrates into the diffuse psychic field. TPQ incorporates *temporal dynamics where subjective time emerges from quanta superposition collapse*, unifying linear neural processing with atemporal field states. This model integrates recent updates to contemporary informational panpsychism, the Orchestrated Objective Reduction (Orch-OR) theory and Integrated Information Theory (IIT), predicting specific neurophilosophical correlates such as gamma synchronization (40–100 Hz) and inter-individual affective resonance. *Predictions include EEG protocols for NDE gamma surges and past-life memory imprints in children.* The theoretical implications include reinterpretation of psychological wellbeing as maximum phase coherence between the individual quantum and the universal field, explanation of non-local emotional communication between minds, and empirically verifiable predictions regarding neural patterns during empathic interactions, NDEs, *telepathic intuition*, and post-mortem awareness. Finally, TPQ proposes a resolution to Chalmers’ “hard problem” of consciousness (1995) through dual ontology integrating physical-material and psychic-informational domains.

## 1 Introduction

### 1.1 The problem of consciousness in contemporary science

The scientific understanding of consciousness remains one of the most profound challenges facing human thought. Despite significant advances in cognitive neuroscience, neuroimaging, and experimental psychology, the fundamental problem of subjective experience—how physical–chemical neurobiological processes generate conscious perception of self and world—remains substantially unresolved. This gap between objectively observable neural activity and subjective phenomenal experience has been termed the “hard problem” of consciousness and continues to challenge traditional reductionist explanations.

Current neuroscientific theories describe with increasing precision the neural correlates of consciousness (NCC), identifying brain configurations associated with conscious states. However, the causal nexus between neural activity and subjective phenomenology remains conceptually opaque. The purely emergentist approach, which views consciousness as a byproduct of neural complexity, proves insufficient from an explanatory standpoint. Simultaneously, classical dualist positions do not offer a coherent and falsifiable operational model capable of integrating the mental and physical dimensions.

In response to this theoretical impasse, alternative perspectives have emerged in recent decades proposing to treat consciousness as a fundamental property of physical reality rather than as an epiphenomenon of matter. Among these are contemporary panpsychism, Integrated Information Theory (IIT), and the Orchestrated Objective Reduction (Orch-OR) hypothesis. These theories, while diverging in implementation details, share the principle that consciousness derives from universal physical properties still partially unknown, linked to the deep structure of information and quantum mechanics.

#### 1.1.1 Connection to quantum theory fundamentals and psychic quanta-qubits relationship

The Theory of Psychic Quanta (TPQ) grounds in quantum theory fundamentals—wave-particle duality, superposition, and entanglement. Psychic quanta are proposed as informational qubits (quantum bits) within a non-local psychic field, analogous to qubits in quantum computing: each psychic quantum encodes conscious experience as a superposition of informational states |ψ⟩ = α|0⟩ + β|1⟩, collapsing via interaction with neural gamma synchronization (40–100 Hz). This establishes TPQ as a quantum-informational bridge between physical and phenomenal domains, integrating formal quantum mechanics (Nielsen and Chuang, 2010) with consciousness studies (Hameroff and Penrose, 2014). The psychic qubit represents the fundamental unit of experiential information, analogous to classical computation qubits but encoding phenomenal rather than algorithmic states.

### 1.2 Motivation and purpose of the theory of psychic quanta

Within this theoretical context lies the Theory of Psychic Quanta (TPQ), which proposes the existence of a fundamental form of non-local energy and information—termed psychic radiation—of which every conscious manifestation represents a localized and individualized condensation. Such a psychic field, hypothesized as coextensive with the universe, would constitute the unifying ontological substrate between mind and matter, offering a possible path beyond the historical division between physicalism and phenomenology.

The central objective of TPQ is not merely to describe consciousness in terms compatible with current physical models, but to propose a new ontology of subjectivity in which the conscious unit—the informational quantum—represents the elementary quantum of the universal psychic field. This paradigm opens the way to an interdisciplinary reformulation involving theoretical physics, neuroscience, cognitive psychology, and philosophy of mind, in the perspective of an integrated model of psychic reality and its biological manifestation.

## 2 Theoretical foundations of the theory of psychic quanta

### 2.1 The universal psychic field

TPQ postulates the existence of a universal non-local psychic field, as fundamental as the electromagnetic field or quantum scalar field. Such a field would permeate the entire universe and constitute the primary ontological substrate from which individual conscious phenomena emerge.

Unlike traditional physical fields, the psychic field does not transport energy-momentum in the classical sense but rather transports experiential information—that is, the potentiality for states of consciousness to arise. This characteristic makes it analogous to quantum fields in relativistic field theory, in which particles are localized excitations of the same field.

The properties attributed to the psychic field are the following:

1. *Non-locality*: It extends throughout space–time without apparent limit;
2. *Informationality*: It carries information structures capable of generating consciousness;
3. *Self-organization*: It exhibits a natural propensity toward coherent and ordered states;
4. *Subjective potentiality*: It contains the ontological potentiality of every form of conscious experience.

### 2.2 The informational quantum: the quantum of consciousness

The central hypothesis of TPQ postulates the existence of an elementary entity of psychic reality, termed the informational quantum (or Psychotone). It represents the quantum of consciousness—the smallest indivisible unit of subjective experience and psychic information capable of anchoring to biological systems of varying complexity—from the human brain to animal organisms, insects, plants, and even microorganisms—manifesting differentiated degrees of phenomenal awareness.

The presence of the informational quantum does not require advanced neural complexity: interaction can occur with elementary biological systems exhibiting adaptive responses to the environment (plant tropisms, primordial behaviors in insects, coordinated biochemical reactions in microorganisms). These manifestations—fear, courtship, orientation, defense—are not devoid-of-subjectivity automatisms, but phenomenological evidence of an anchored quantum acting as spectator and actor of that biological center of consciousness.

In analogy with the photon, which constitutes the quantum of electromagnetic radiation, the informational quantum is conceived as the fundamental quantum of universal psychic radiation. A more complex brain offers the quantum more sophisticated experiential tools, but consciousness does not emerge from neural complexity. The quantum exists independently and modulates the depth of its manifestation in proportion to the complexity of the available biological substrate, always maintaining its unitary and fundamental nature.

The informational quantum possesses the following defining properties:

- *Unitarity*: It is indivisible and cannot be decomposed into smaller units of consciousness;
- *Coherence*: It maintains a stable quantum superposition state until anchoring;
- *Non-materiality*: It possesses no mass, electric charge, or other measurable material properties;
- *Informationality*: It is defined by its informational coherence properties and phenomenal presence;
- *Anchorability*: It can bind to coherent brain systems, acquiring spatiotemporal localization and individuality.

#### 2.2.1 Formal propositions of TPQ

*Proposition 1*: Psychic quanta exist as quantized informational excitations in a universal psychic field (Ψ_field). Each psychic quantum represents a discrete, indivisible unit of conscious information, ontologically fundamental and non-emergent from matter.

*Proposition 2*: Brain psychotones (coherent neural assemblies operating at gamma frequencies 40–100 Hz) function as bidirectional interfaces, entangling physical qubits (neural coherence) with psychic qubits (conscious information) via thalamocortical gamma-band coherence and microtubular quantum effects.

*Proposition 3*: Conscious experience emerges from collapse of psychic qubit superposition during psychotone-field interaction. This collapse is triggered by neural coherence thresholds, yielding subjective phenomenal awareness as the experienced correlate of qubit state reduction (Hameroff and Penrose, 2014).

#### 2.2.2 The wave-particle duality of the psyche

TPQ extends the principle of complementarity from quantum mechanics to the psychic domain. The informational quantum manifests a functional duality:

1. *Diffuse state (wave)*: In the absence of binding to a biological system, the quantum manifests as diffuse psychic radiation, a non-localized field state lacking subjective individuality;
2. *Localized state (particle)*: In the presence of a mature and functioning brain, it collapses into a localized unit of consciousness, acquiring a defined subjective point of view and persistent experiential identity.

This process can be formally represented as a state transition in the psychic field.

This state remains coherent and unitary throughout the duration of biological life, until the cessation of brain activity, at which point the anchoring dissolves and the quantum spontaneously returns to the state of diffuse non-localized psychic radiation.

### 2.3 The interaction of the psychic quanta with the brain

#### 2.3.1 The mechanism of quantum-psychic anchoring

According to TPQ, individual consciousness emerges from the dynamic interaction between the informational quantum—the elementary unit of the psychic field—and the brain system, which serves as a reciprocal biophysical interface. This interaction does not imply consciousness generation by the brain in an emergentist sense, but rather a process of anchoring and modulation: the brain acts as a receiver-transducer capable of structuring quantum activity into coherent and adaptively functional mental contents.

TPQ hypothesizes that the interaction between the quantum and the biological brain occurs through a process of quantum-informational resonance, founded on coherence states present in specific neurophysiological structures.

Quantum anchoring requires a minimum threshold of structural and informational coherence in the biological system, not necessarily a complex brain. This threshold is observable in:

1. Animal organisms: Intentional behaviors, fear, play, individual recognition;
2. Insects: Navigation, associative learning, pheromone communication;
3. Plants: Photosynthetic tropisms, chemical defensive responses, electrical signaling;
4. Microorganisms: Chemotaxis, quorum sensing, biochemical memory.

The distinction between anchored quantum and diffuse psychic field becomes subtle in elementary biological systems, suggesting a continuum of partial anchoring rather than a sharp dichotomy.

The human brain cannot be described exclusively in terms of classical neurochemical processes. Contemporary models, such as the Orchestrated Objective Reduction (Orch-OR) proposed by Penrose and Hameroff, suggest that in neuronal microtubules—cylindrical structures of the cellular cytoskeleton—coherent quantum states may be established capable of influencing synaptic function and global neural dynamics.

Such quantum states present temporal coherence times compatible with the dynamics of brain gamma rhythms (40–100 Hz), which neurophysiology associates with unitary perceptual integration and the manifestation of consciousness. Similarly, the theory of electromagnetic coherence domains developed by Del Giudice et al. (1988) shows that in biological systems water and biomolecules can enter collective oscillatory states, establishing regions of persistent quantum resonance. These coherent areas, particularly abundant in the brain, would constitute the physical substrate most suited to interfacing with the universal psychic field.

Quantum anchoring in complex neural systems requires that the brain system reach a configuration of dynamic complexity and informational coherence sufficient to establish a stable resonance window with the psychic field. This state would typically occur at the end of fetal development or in conjunction with biological birth, the moment when the neural network acquires a global oscillatory structure capable of supporting thalamocortical synchronization.

The interaction can be formalized through a Hamiltonian coupling function between the two fields, yielding:

When the two wave functions are in phase (constructive resonance), the interaction is maximal and the state of unitary consciousness is generated, resulting from the coherent superposition between the physical and psychic domains.

Well-documented brain phenomena from the neuroscientific literature could represent indirect empirical traces of such psychic-physical coupling:

- Cortical gamma synchronization (40–100 Hz): Correlated with unitary perceptual integration and associated with conscious awareness states;
- Thalamocortical resonance: Which maintains the continuity of conscious experience during wakefulness;
- Coherent oscillatory activity of microtubules: Recently observed *in vitro* in neuronal preparations;
- Dynamics of cortical electromagnetic fields: Which could constitute the physical channel through which the quantum modulates neural activity.

#### 2.3.2 Bidirectionality of coupling

The interaction between quantum and brain is not unidirectional but reciprocal:

1. *Psychic afference*: The quantum receives sensory information processed by the neural network and translates it into subjective conscious experience, endowing it with meaning and intentionality;
2. *Psychic efference*: The quantum returns to the brain a stream of intentional modulation that manifests as selective attention, volitional decision, intentional action, and will.

The cessation of brain activity causes the loss of structural coherence necessary for anchoring, with consequent spontaneous disanchoring of the quantum, which returns to the diffuse state of the psychic field. This transition can be interpreted as the dissolution of the individual self into the continuum of universal consciousness, coherently with the ontological description introduced in previous sections.

### 2.4 Nature and origin of the psychic field

#### 2.4.1 Structure and ontological properties

The central hypothesis of TPQ predicts that the universe is not constituted solely of fields of energy and matter—as postulated by contemporary scientific physicalism—but is instead an ontologically dual system, permeated by a fundamental psychic field from which individual conscious phenomena emerge.

Such a field would represent the subjective counterpart of the cosmos, analogous in ontological role to what the quantum scalar or vector field assumes in theoretical physics for the description of matter and radiation.

The psychic field can be conceived as a non-local continuum of conscious information, distributed throughout spacetime and endowed with intrinsic self-organizational potentialities. Unlike traditional physical fields, which transport measurable energy and momentum, the psychic field transports experiential information—that is, the potentiality for states of subjective consciousness to arise.

Analogously to standard quantum mechanics, where material particles are localized excitations of a field, the informational quantum represents the localized excitation of the universal psychic field. When such excitation anchors to a coherent brain system (as described in previous paragraphs), the result is individual subjective experience. When anchoring ceases, the quantum returns to the diffuse field state, dissolving the phenomenological individuality maintained during biological life.

The fundamental question remains whether the psychic field possesses an intrinsic form of individuality, or represents an impersonal and non-conscious matrix from which only emerge as epiphenomena the temporary and localized centers of consciousness.

The observation of deep structural coherence in the universe—the verified stability of fundamental physical constants, the evolutionary directionality toward increasing complexity, and the self-organizational capacity demonstrated by complex biological systems—strongly suggests that the universe is not a random and disordered system but rather is governed by intelligible and directed organizing principles.

From this perspective, the psychic field could constitute the fundamental intentional principle of the universe, that is, the cosmological source from which both material order and the intrinsic tendency toward complexity and consciousness derive. This vision finds profound resonance in the panpsychic and cosmopsychic hypotheses developed by contemporary philosophy of mind, according to which consciousness does not emerge from inert matter through evolutionary contingencies but rather is a fundamental and ubiquitous property of the cosmos itself.

Within the context of TPQ, the informational quantum represents the ontological mode through which this universal consciousness individualizes, temporarily assuming a localized subjective perspective. The empirical individual self would thus not be the product generated by the brain but rather the result of spatial and temporal limitation of universal consciousness, made possible by the coherent brain interface.

#### 2.4.2 Individuality and universal psychic consciousness

TPQ predicts the existence of “free Psychotones”—psychic units not anchored to biological substrates—awaiting first anchoring or subsequent biological reincarnations. These units would maintain residual emotional connections with quanta previously interacted with during shared biological lives, explaining phenomena of instantaneous recognition and profound affinities between individuals meeting for the first time.

This hypothesis introduces the possibility of a “relational mnemonic field” in which free units preserve informational traces of previous interactions, constituting the theoretical basis for concepts such as “twin souls,” relational karma, and ancestral memory.

Such connections would persist despite biological disanchoring, maintaining emotional coherence among units in a state of non-locality and permitting forms of affective communication even without brain intermediation.

#### 2.4.3 Indirect evidence and theoretical parallels

Several streams of contemporary research provide conceptual clues compatible with the existence of a diffuse psychic principle coextensive with the cosmos:

1. *Integrated Information Theory (IIT)*: Developments in Tononi’s theory show that consciousness increases monotonically with the amount of unified information (*Φ*) present in a system. This suggests that information itself possesses intrinsic phenomenic qualities;
2. *Self-organization models:* Works by Prigogine on systems far from equilibrium and by Kauffman on autocatalytic networks demonstrate that matter spontaneously tends toward states of greater complexity and coherence, in apparent ordered evolutionary direction;
3. *Cosmopsychic philosophical reflections:* Classical authors such as Whitehead and Teilhard de Chardin anticipated more than a century ago the possibility that consciousness is the internal and subjective dimension of the cosmic evolutionary process, not its random epiphenomenon.

These heterogeneous contributions converge in delineating an image of the universe endowed with intrinsic intelligibility and self-organization, in which matter appears as the external and objective aspect of a deeper principle of psychic and informational nature.

## 3 Empirical predictions and theoretical implications

### 3.1 Psychic resonance as the foundation of wellbeing

#### 3.1.1 Mathematical model of resonance

A natural and coherent extension of the Theory of Psychic Quanta concerns how informational quanta interact not only with their own biological brain substrate but also with each other and with the universal psychic field.

Since the quantum represents a localized excitation of a unitary field coextensive with the universe, every variation in the state and vibrational frequency of one individual quantum influences—albeit minimally through field propagation effects—the entire psychic field and, by necessary consequence, all other psychic units present.

From this theoretical perspective, the subjective experience of psychological wellbeing can be interpreted not as a simple abstract psychological variable but rather as a phenomenological indicator of the degree of coherence and resonance between the state of the individual quantum and the global vibrational frequency of the universal psychic field.

The condition of wellbeing can be formally described as a maximum of phase correlation between the state of the individual quantum and the average state of the universal psychic field, with mathematical expression showing that when perfect alignment occurs, the system reaches maximum resonance and the subjective sensation is one of inner harmony, profound equilibrium, and complete psychosomatic wellbeing. As phase dissonance increases, the perceived psychological distress and emotional discomfort increase proportionally.

Conversely, when the actions, intentions, and emotional states of an individual are in harmonious synchronization with the organizing directionality of the universal psychic field, the quantum experiences a condition of maximum resonance, phenomenologically perceived as inner harmony, profound equilibrium, and integral wellbeing. Conversely, when individual behavior deviates significantly from such coherence—generating internal conflict, relational disharmony, or disconnection from other quanta—there spontaneously emerge states of emotional distress, cognitive discomfort, and physical discomfort, as the neurophysiological expression of reduced psychic coherence.

#### 3.1.2 Verifiable neurophysiological correlates

This model is coherent with several consolidated neuroscientific and psychological evidences demonstrating:

- *Neuronal coherence and positive emotional states:* Neuroimaging studies show correlation between brain gamma synchronization and positive emotional states of wellbeing;
- *Empathic attunement and wellbeing:* Literature on emotional regulation demonstrates the importance of empathic attunement and social regulation in determining overall wellbeing;
- *Physiological harmony and states of calm:* Research on heart rate variability and respiratory coherence documents the correlation between physiological harmony and mental states of calm, inner centering, and increased awareness.

These empirical observations suggest that psychological wellbeing is not a simple abstract psychological epiphenomenon but rather the concrete phenomenal manifestation of profound and verifiable resonance between the individual quantum and the universal psychic field.

#### 3.1.3 Evolutionary and social implications

In terms of evolutionary biology and adaptation, this model could explain why cooperative, empathic, and altruistic conduct is statistically associated with greater psychosomatic equilibrium, better mental health, and greater social stability. Such conduct maintains psychic phase coherence between living beings, stabilizing the entire field and generating conditions of individual and collective wellbeing.

### 3.2 Psychic and emotional communication between quanta

#### 3.2.1 Direct non-local communication

TPQ hypothesizes that communication between quanta and between these and the universal psychic field occurs through an intrinsic emotional language, that is, a form of informational exchange based on primary affective states rather than on logical symbols or elaborate conceptual structures.

In biological species endowed with superior cognitive complexity, such as humans, this primary emotional language is filtered, modulated, and transduced into “superstructured” systems of communication anchored to biological matter—articulated verbal language, bodily and behavioral expressions, chemical and olfactory communication, vocal and gestural signals.

In this evolutionary sense, emotional activity represents the primary translation channel through which the quantum expresses itself and consciously interacts with the physical environment and with other incarnate quanta.

Humans, as quanta incarnate in a biologically complex nervous system, learn not only to reason logically and manipulate symbols but also to interact emotionally, correlating their own emotions with those of others through superstructured and conventional languages typical of the species.

However, everyday phenomenological experience shows that direct emotional connection can occur even in the absence of explicit conventional languages or in conditions of significant physical distance. Well-documented phenomena such as instantaneous empathy, spontaneous affective synchronization, and the feeling of “invisible connection” between loved ones—particularly between relatives—suggest the existence of a direct and non-local psychic communication channel between quanta, likely mediated by the universal psychic field.

This type of emotional connection appears to significantly transcend the biological and spatial boundaries of the individual body. Numerous phenomenologically documented testimonies and psychological studies on extrasensory perception, Jungian synchronicity, and affective intuition at a distance suggest the conceptual possibility that psychic communication extends even toward deceased individuals and toward the universal psychic field itself.

#### 3.2.2 Post-mortem continuity and gradual dissolution

This would imply significant ontological implications: after the quantum detaches from the deceased brain, it would temporarily maintain its individual identity and coherent structure, continuing to exist in a state of emotional coherence with incarnate quanta and with the universal psychic field.

In such a cosmopsychic scenario, the phase following biological disanchoring would not entail immediate and total dissolution into the undifferentiated state of the universal field but rather a gradual and temporally extended fusion, during which the quantum would temporarily preserve the capacity to communicate and interact at the emotional level with still-incarnate quanta.

This theoretical hypothesis opens fascinating new perspectives on the continuity of individual consciousness beyond biological death, on the non-local and fundamental nature of affective experience, and on the possibility of integrating subjective phenomenology, cognitive neuroscience, and quantum field physics into a single coherent and potentially falsifiable framework.

### 3.3 Verifiable predictions

TPQ advances several empirically verifiable predictions that could constitute the foundation of future research programs:

1. *Neural coherence patterns during empathic interactions:* EEG and fMRI measurements during empathic interactions should reveal inter-brain gamma synchronization and increased phase coherence between subjects in profound emotional contact;
2. *Neural correlates of wellbeing:* Neuroimaging during states of psychological wellbeing should show increased synchronization among regional brain rhythms and dominance of gamma frequencies;
3. *Near-death experience phenomena:* Studies of NDEs should reveal coherent brain activity patterns despite cessation of cerebral circulation, suggesting persistence of psychic state during biological disanchoring;
4. *Post-mortem experiential continuity*: Prospective studies of post-mortem experiential testimonies should reveal consistent patterns of descriptions of emotional communication with deceased individuals.

## 4 Discussion

### 4.1 Strengths of the theory

#### 4.1.1 Ontological coherence and falsifiability

TPQ’s primary strength lies in its falsifiability via EEG/fMRI tests of gamma synchronization (40–100 Hz) during near-death experience induction or deep meditative states, predicting heightened quantum coherence patterns that can be quantitatively measured (Hameroff and Penrose, 2014 updates; Borjigin et al., 2013). Unlike purely emergentist models, which lack empirical specificity, TPQ proposes testable predictions on neural correlates that directly relate to conscious experience. The theory avoids neurocentrism and Cartesian dualism by proposing a dual but integrated ontology of the physical and psychic domains.

#### 4.1.2 Mathematical formalism and quantum integration

TPQ proposes concrete mathematical formulations (Hamiltonian coupling, wave function collapse dynamics, phase coherence metrics) that can be refined toward greater precision and computational modeling. The theory integrates established quantum theory (superposition, entanglement, measurement problem) with neuroscience, creating a coherent framework where psychological phenomena are grounded in quantum principles rather than classical emergence.

#### 4.1.3 Philosophical integration: Žižek’s quantum ontology

From an ontological perspective, philosopher Slavoj Žižek’s recent work “Quantum History: A New Materialist Philosophy” (2025) complements TPQ by exploring quantum contingency as a fundamental feature of subjective experience. Žižek argues that consciousness emerges not from predetermined physical laws but from quantum indeterminacy itself—a position perfectly aligned with TPQ’s proposition that psychic quanta mediate between quantum potentiality and phenomenological necessity (Žižek, 2025). This integration grounds TPQ in contemporary Continental philosophy, strengthening its philosophical rigor beyond purely empirical neuroscience.

#### 4.1.4 Temporal dynamics in TPQ

One critical dimension previously underdeveloped is the conceptualization of time within TPQ. We here propose: Time in TPQ emerges dynamically from psychic quanta superposition collapse, yielding subjective ‘arrow of time’ via sequential quantum-field interactions and neural state transitions (inspired by Vitiello’s dissipative quantum field models of brain dynamics; Vitiello, 2001). The linear perception of time at the neural level (synaptic transmission ~milliseconds, neural oscillations ~tens of milliseconds) reflects the sequential collapse of superposition states as consciousness samples informational moments.

The universal psychic field, by contrast, exhibits atemporal properties—all possible states exist in superposition—until localized interaction with a brain system yields experiential temporality. Crucially, the non-local character of the field theoretically enables retrocausal effects (precognition), where information flows backward in time via quantum entanglement patterns. This can be empirically tested via pre-stimulus EEG protocols measuring intuitive anticipation of random stimuli (McCraty, 2014; Radin et al., 2012). Thus, TPQ unifies linear neural time (classical information processing) with atemporal consciousness field dynamics (quantum superposition), explaining both forward causality in ordinary experience and backward causality in paranormal phenomena.

#### 4.1.5 Empirical predictions and testable protocols

To advance TPQ from theory to empirically grounded science, we propose concrete testable protocols:

*H1*: Psychotone Coherence and Gamma Synchronization: Test via 64-channel EEG during meditation induction and NDE cardiac arrest protocols—predict statistically significant 20–30% increase in gamma power (40–100 Hz) correlating with reported meta-consciousness and expanded awareness (Borjigin et al., 2013, study of anesthetized rats showing gamma surge during brain shutdown; Parnia et al., 2014 prospective cardiac arrest studies). Null hypothesis: No significant gamma increase predicts either TPQ requires revision or classical neural mechanisms dominate post-mortem consciousness.

*H2*: Bidirectional Field-Brain Interface in Lucid Dreams: fMRI studies of lucid dreamers during REM sleep should show: (a) sustained high-frequency gamma coherence despite reduced external sensory input, and (b) prefrontal-hippocampal-parietal connectivity patterns consistent with internal field-generation rather than stimulus-driven response (Voss et al., 2025, recent meta-analysis). This tests whether brain generates consciousness (classical prediction: reduced sensory input → reduced consciousness) or modulates pre-existing psychic field (TPQ prediction: consciousness remains high despite sensory isolation).

*H3*: Inter-brain Coherence and Empathic Resonance: Simultaneous dual-EEG from pairs in deep empathic dialogue should reveal inter-individual gamma synchronization (phase coupling at 40–100 Hz) proportional to empathy rating scales and emotional closeness. This directly tests whether psychic field mediates affective communication via field resonance rather than solely through verbal/chemical signals (Dumas et al., 2010 preliminary evidence of inter-brain synchrony during mother-infant interactions).

*H4*: Pre-stimulus Intuition as Retrocausal Field Effect: EEG and skin conductance recordings during random stimulus anticipation tasks (Radin et al., 2012) should show statistically significant pre-stimulus physiological responses (100–500 ms before stimulus), inconsistent with classical causality but consistent with backward-time field effects. This directly tests retrocausality as predicted by TPQ's non-local psychic field.

#### 4.1.6 Parapsychological phenomena and TPQ applications

Telepathic Intuition as Quanta Entanglement: TPQ explains spontaneous telepathic phenomena—emotional resonance between distant individuals without conventional communication—as direct psychic quanta entanglement. When two individuals have shared history or emotional intimacy, their quanta become entangled at the field level, permitting non-local information exchange. Meta-analytical evidence from decades of telepathy experiments reveals effect sizes of p ≈ 10^-7, far exceeding chance (Sheldrake, 2008, 2011; Bem and Honorton, 1994). TPQ provides ontological grounding: telepathy represents field-level resonance between psychic qubits, analogous to quantum entanglement in particles, not a mystical phenomenon but a natural consequence of unified psychic field theory.

Precognition as Retrocausal Field Effect: Precognitive experiences—"knowing” future events before they occur—follow naturally from TPQ’s non-local, atemporal psychic field. Information propagates through the field backward and forward in time via quantum entanglement. While precognition appears paradoxical in classical physics, it becomes logically consistent in quantum field theory where temporal ordering of causally disconnected events is not absolute. Experimental evidence (Radin et al., 2012; Bem’s presentiment studies, 2011) shows humans physiologically respond to random future stimuli ~300 ms before stimulus occurs, consistent with retrocausal information flow.

Reincarnation and Persistent Psychic Quanta Imprints: TPQ’s “free psychic units” hypothesis proposes that after brain death, the informational quantum disanchors but persists in the universal field, retaining informational imprints of previous experiences. This provides theoretical basis for documented cases of past-life memory in children. The University of Virginia’s Division of Perceptual Studies (DOPS) has catalogued over 3,000 cases where young children (2–6 years) spontaneously recall detailed memories of previous lives (names, places, cause of death), many verified through independent investigation (Tucker, 2005; Stevenson, 1974). A follow-up study of 1,400 children with past-life memories found 65% experienced lifelong psychological impact and higher-than-normal psychological functioning, inconsistent with false memory hypothesis but consistent with persistent quantum imprint theory (Tucker, 2005). While controversial, TPQ grounds these phenomena in quantum informational persistence rather than mysticism or fraud.

### 4.2 Limitations and open challenges

Quantum-Physical Validation: The quantum-physical arguments presented in TPQ are theoretical and should be validated by domain experts in quantum physics through rigorous empirical testing and mathematical refinement. While the framework draws on established quantum principles (superposition, entanglement, measurement collapse), its application to consciousness remains speculative. Collaboration with quantum physicists is essential to ensure physical consistency.

1. *Direct Quantum Verifiability*: The informational quantum is not directly measurable with current technology; research must proceed through indirect neurophysiological correlates (EEG gamma, fMRI BOLD, etc.). Future quantum sensors capable of measuring entanglement patterns might enable direct detection.
2. *Complete Mathematical Formalization*: The proposed Hamiltonian formalism requires further development toward a rigorous quantum-relativistic formulation, including Lorentz covariance and consistency with special relativity.
3. *Integration with Computational Models*: Numerical simulations of the coupling function are necessary to test quantitative predictions during various consciousness states (meditation, sleep, anesthesia, NDE).
4. *New Experimental Methodologies*: Novel research protocols are necessary to rigorously investigate phenomena such as post-mortem continuity, past-life memories, and extrasensory perception in controlled laboratory settings while maintaining scientific objectivity.

### 4.3 Future research perspectives

Future research should be systematically oriented toward addressing these limitations and testing TPQ predictions:

1. *Multi-modal Neuroimaging Programs*: EEG/fMRI/MEG studies during profound meditation, lucid dreaming, and prospectively during near-death experiences in cardiac units to detect gamma synchronization patterns and inter-brain synchronization.
2. *Computational Modeling and Simulation*: Development of numerical simulations of the Hamiltonian coupling equation to predict consciousness dynamics across various brain states and test against empirical data.
3. *Interdisciplinary Collaboration*: Partnerships between theoretical physicists (quantum mechanics expertise), neuroscientists (brain imaging), philosophers of mind (ontological rigor), and consciousness researchers.
4. *Rigorous Parapsychology Research*: Prospective studies on past-life memory, telepathic phenomena, and precognition using contemporary neuroscience methods while maintaining methodological rigor and scientific openness.
5. *Quantum Sensor Development*: Investment in next-generation quantum sensors capable of detecting non-local field effects, entanglement patterns, and subtle electromagnetic coherence domains.
6. *Integration with Current Models*: Formal comparative analysis with Integrated Information Theory, Global Workspace Theory, Higher-Order Thought theories, and Orch-OR.

## 5 Conclusion

The Theory of Psychic Quanta reformulates the problem of consciousness beginning from a radically new perspective: consciousness not as an epiphenomenon of neural complexity but as quantization of a fundamental universal psychic field. In this dual ontology, the brain does not generate consciousness but acts as a dynamic interface that stabilizes and modulates the informational quantum—the quantum of consciousness—permitting its temporary biological incarnation.

The theoretical implications are profound: psychological wellbeing is not a simple psychological state but the phenomenal reflection of profound resonance between the individual and the universal psychic field. Biological death does not entail the annihilation of consciousness but rather its disanchoring from the brain substrate and its gradual reintegration into the non-local psychic domain. Time itself emerges from quantum collapse dynamics, unifying subjective experience with physical evolution.

Although not yet completely formalized mathematically and requiring further experimental verification, TPQ offers a coherent, multidisciplinary, and potentially falsifiable theoretical framework for addressing Chalmers’ “hard problem” of consciousness. It integrates scientific rigor, philosophical sophistication (particularly through integration with Žižek’s quantum ontology), and respectful engagement with paranormal phenomena documented across cultures and centuries.

Future research, through advanced neuroimaging (EEG/fMRI protocols), computational modeling (Hamiltonian simulations), interdisciplinary studies (physics-neuroscience-philosophy collaboration), and rigorous investigation of consciousness boundary phenomena (NDEs, past-life memories, telepathy, precognition), can subject TPQ to rigorous empirical testing. This transformative theoretical hypothesis has potential to evolve into a solid and enduring scientific contribution to understanding the deepest nature of consciousness and the universe.

## Data Availability

The original contributions presented in the study are included in the article/supplementary material, further inquiries can be directed to the corresponding author.
